# A pangenome-based strategy for designing a multi-epitope vaccine against non-toxin antigens of necrotic enteritis-associated *Clostridium perfringens*

**DOI:** 10.1007/s42770-026-01968-y

**Published:** 2026-05-13

**Authors:** João Pedro Gomes Greco, Chrystian Nunes Gonçalves, Fabricio Rochedo Conceição, Frederico Schmitt Kremer, Luciano da Silva Pinto

**Affiliations:** Centro de Desenvolvimento Tecnológico, Universidade Federal de Pelotas, Capão do Leão, Rio Grande do Sul Brazil

**Keywords:** Immunoinformatics, Reverse vaccinology, Virulence factors, Poultry health, Pangenomics, Synthetic biology

## Abstract

Avian necrotic enteritis (NE), caused by *Clostridium perfringens*, poses a significant threat to the global poultry industry, exacerbated by rising antibiotic resistance. In this study, we applied a pangenome-guided reverse vaccinology approach to design a multi-epitope chimeric vaccine candidate. From the analysis of 45 genomes, our filtering pipeline identified three conserved and functionally synergistic target proteins: the type IV pilus assembly protein PilO and the prepilin peptidase-dependent protein A, both necessary for bacterial adhesion, and the regulatory protein MsrR, which is crucial for cell-wall integrity. High-affinity B-cell epitopes derived from these three proteins were assembled into an optimized chimeric construct. Structural modeling and molecular docking with *Gallus gallus* Toll-like receptors (TLRs) indicated strong binding affinity, while physicochemical analyses predicted high stability and antigenicity. We also propose an experimental validation plan encompassing recombinant production, formulation timeline, and in vivo endpoints in chickens, aligned with protein-based MEVs that have demonstrated efficacy in bacterial models. This work presents a rationally designed vaccine candidate that simultaneously targets the pathogen’s offensive (adhesion) and defensive (cell integrity) mechanisms, offering a robust computational framework to accelerate the development of antibiotic alternatives in poultry production.

**Supplementary Information:**

The online version contains supplementary material available at 10.1007/s42770-026-01968-y.

## Introduction

Avian Necrotic Enteritis (NE), caused by *Clostridium perfringens*, is a globally distributed disease that primarily affects poultry, especially broiler chickens. It is estimated to cause annual losses of approximately USD 6 billion worldwide [1]. As the demand for animal-derived products increases and the global livestock sector continues to expand, these losses are expected to grow proportionally. The disease is characterized by severe damage to the intestinal mucosa, reduced weight gain, decreased feed intake, low growth rate, diarrhea, and weakness, with mortality rates ranging from 2% to 50%. NE mainly affects chickens between 2 and 6 weeks of age [2]. *C. perfringens* is an anaerobic, spore-forming bacterium commonly found in the gastrointestinal tract of humans and other animals, as well as in soil and decaying organic matter [3]. It is currently classified into seven types (A–G) based on toxin production, which are responsible for distinct disease manifestations in both humans and animals. These toxins include alpha (α), beta (β), epsilon (ε), iota (ι), enterotoxin (CPE), and NetB. The alpha toxin (CPA) is the only one present in all *C. perfringens* strains, whereas the others are plasmid-encoded: beta toxin (CPB, *cpb* gene), epsilon toxin (ETX, *etx* gene), iota toxin (ITX, *iap* and *ibp* genes), enterotoxin (CPE, *cpe* gene), and NetB (*netB* gene). The recent classification expansion includes types F and G, corresponding respectively to CPE-producing strains associated with human food poisoning and NetB-producing strains responsible for necrotic enteritis in poultry [4].

Necrotic enteritis is widely distributed across poultry-producing regions worldwide, with reported prevalence ranging from 1% to 40%, affecting a substantial proportion of commercial broiler flocks depending on production systems and management practices [1, 5, 6]. Traditionally, vaccine development against NE has focused on toxin-based antigens, particularly CPA and NetB, which are key virulence factors involved in disease pathogenesis. Several experimental and commercial approaches, including toxoid-based vaccines and recombinant protein formulations targeting these toxins, have been investigated. However, despite its economic importance, effective control through vaccination remains limited. These strategies have generally provided only partial protection, primarily reducing disease severity rather than preventing bacterial colonization and infection [7, 8].

Antibiotics have historically been used to control NE and improve animal performance by modulating gut microbiota and suppressing pathogenic bacteria, thereby improving animal health, nutrient availability, and growth [9]. However, the widespread use of antimicrobial drugs has contributed to the emergence of antibiotic-resistant strains, leading to regulatory restrictions on their use in animal production associated with public health risks [10]. Consequently, alternative strategies such as vaccination, prebiotics, and probiotics have gained increasing attention as sustainable approaches to disease control [11].

Vaccines have been developed using diverse approaches, including inactivated or attenuated microorganisms, recombinant DNA or RNA platforms, glycoconjugates, and adjuvants, and remain essential tools to combat antibiotic-resistant bacteria. By priming the host immune system, vaccination prevents infection upon exposure and, unlike antibiotics that act on single molecular targets, elicits multifaceted immune responses that reduce the likelihood of resistance development [12]. Advances in genomic sequencing have further transformed vaccine design through reverse vaccinology, which enables the in silico screening of microbial genomes to identify antigens likely to be surface-exposed or secreted [13]. These candidates can then be selected, recombinantly expressed, commonly in *Escherichia coli*, and evaluated in preclinical models [12], providing a rational and targeted strategy to accelerate vaccine development.

Despite progress in toxin-based vaccines, evidence from *Clostridioides difficile* studies indicates that targeting toxins alone may not prevent bacterial colonization or primary infection. Recent phase III clinical trials of toxoid-based vaccines failed to achieve primary endpoints despite strong antitoxin responses [7, 8]. These findings highlight the importance of incorporating non-toxin antigens, particularly those involved in bacterial adhesion and colonization. Surface-associated proteins have shown promise in inducing both mucosal and systemic immunity, suggesting that targeting colonization mechanisms may enhance vaccine efficacy. In this context, the present study applies a pangenome-guided reverse vaccinology approach to identify conserved, functionally relevant antigens and design a multi-epitope vaccine candidate against NE.

## Materials and methods

### Data collection

The data were collected from NCBI BioSample using the following criteria: (A) samples derived from *Gallus gallus* and (B) the presence of *C. perfringens* and necrotic enteritis being characterized in the genome. After selection, the genomes were annotated according to strain, ID, and GenBank accession number. The genomes of *C. perfringens* strains related to avian necrotic enteritis, presented in Supplementary Data 1, were retrieved from GenBank and downloaded using the NCBI Datasets command-line tool.

### Pangenome analysis

Genomes of *C. perfringens* associated with necrotic enteritis in *G. gallus* were retrieved from the NCBI database (https://www.ncbi.nlm.nih.gov/datasets/*)*, filtered to remove duplicated entries, and standardized for analysis. The pangenome was constructed using Roary [14] with default parameters, following the conversion of GenBank files into GFF3 format. The core genome was defined as ortholog groups present in at least 95% of the analyzed strains. Representative sequences for each ortholog group were selected for subsequent analyses. The pangenome nucleotide sequences were translated into amino acid sequences to generate a complete proteome dataset, which was partitioned into smaller subsets to ensure compatibility with immunoinformatics tools.

### Immunoinformatics

The translated proteome was screened to identify potential vaccine targets, focusing on secreted and membrane-associated proteins, particularly those related to virulence and pathogenesis. Subcellular localization was predicted with PSORT-B [15], configured for Bacteria and Gram-positive organisms, while DeepTMHMM [16] was applied under default parameters to detect transmembrane helices, and InterProScan was run with its default settings for functional domain annotation. Only proteins conserved in at least 95% of the analyzed strains and fulfilling these criteria were retained as candidates. The resulting dataset was further compared against the Virulence Factor Database (VFDB) [17] using BLAST [18, 19], a specialized database of bacterial pathogen virulence factors, providing detailed information on these factors, including shared genes, functions, and mechanisms of action, and proteins with ≥ 30% identity and bit score > 100 to known virulence factors were selected. Antigenicity of the candidate proteins was assessed using VaxiJen (threshold 0.4) [20] and validated with AntigenPro [21]. The combined use of these tools increased the reliability of predictions, reducing the bias inherent to individual algorithms. Only proteins classified as antigenic by both approaches were retained for downstream epitope mapping and chimera design.

### Comparison with the chicken genome

A comprehensive local BLASTP analysis [18, 19] was performed using protein sequences obtained from the filtered VFDB dataset as queries against a custom *G. gallus domesticus* protein database (GenBank accession: GCA_016699485.1). The protein database was first constructed locally by converting the reference genome protein file into a BLAST database using makeblastdb. Local BLASTP alignments were then executed, and the resulting matches were filtered based on stringent criteria: bitscore greater than 100 to ensure high-quality alignments, E-value less than 1 to retain statistically significant hits, and sequence identity greater than 30% to select biologically relevant similarities. The filtered dataset was subsequently analyzed to identify proteins without similarity from the host genome, thereby highlighting exogenous protein candidates suitable for downstream antigenicity prediction and potential vaccine development [22].

### Final selection of protein targets

All proteins were subsequently analyzed using BLAST against UniProt for a deeper identification and functional annotation. To avoid redundancy, duplicate sequences were removed, keeping only one protein per accession code. Finally, a literature review was initiated for the 24 proteins with the highest antigenic potential, consulting Google Patents (https://patents.google.com/*)* and PubMed (https://pubmed.ncbi.nlm.nih.gov/*)* using the terms: protein name, *vaccine*, *diagnostic*, and *target protein*, to gather evidence of the biological activity of these proteins, even if they have not yet been studied in *C. perfringens*.

### Epitope selection, multi-epitope chimera design, and assembly

Epitope selection was performed on the three proteins with the highest antigenicity scores, previously identified using VaxiJen with a threshold of 0.4. The sequences were submitted to the Immune Epitope Database (IEDB) (http://tools.iedb.org/*)* for the prediction of B-cell epitopes (BepiPred), following the standard protocols for computational epitope mapping [23]. Hydrophilicity analysis was also conducted within IEDB to ensure that accessible, surface-exposed epitopes were prioritized. After clustering to remove redundancy, a final dataset of 54 unique epitopes was obtained for downstream analysis.

To evaluate their interaction with host immune receptors, blind docking simulations were performed using ZDOCK [24]. The epitopes were docked against the receptors TLR2 type 1, TLR2 type 2, TLR4, TLR5, and TLR7. Docking scores were used to select epitopes with the highest predicted binding affinity, with TLR2 type 1 and type 2 showing the most promising interactions with *C. perfringens* epitopes.

Based on these results, a multi-epitope chimera was designed by combining the top epitopes with the highest affinity for each receptor, arranged in the order TLR4 + TLR5 + TLR7 + TLR2 type 1 + TLR2 type 2. To improve conformational flexibility, the epitopes were interconnected using GPGPG linkers, which are widely applied to preserve epitope accessibility and facilitate proteasomal processing for MHC presentation [25, 26].

Codon optimization for *Escherichia coli* expression was performed using the VectorBuilder Codon Optimization Tool (https://en.vectorbuilder.com/tool/codon-optimization.html*)*, with the incorporation of restriction sites *Bam*HI, *Hind*III, and *Eco*RI to facilitate cloning. A C-terminal His-tag was included to allow purification by affinity chromatography. The mRNA secondary structure was analyzed using RNAfold (http://rna.tbi.univie.ac.at/cgi-bin/RNAWebSuite/RNAfold.cgi*)* to confirm transcript stability [27]. Finally, the three-dimensional structure of the chimera was predicted using AlphaFold [28] to verify proper folding and overall stability.

### Prediction of antigenicity

To confirm the immunogenic potential of the selected proteins, antigenicity was re-evaluated using the same dual strategy (VaxiJen 2.0 and AntigenPro). This step ensured consistency with the initial screening and confirmed that only proteins with high antigenic scores were advanced to epitope prediction and chimera construction.

### Physicochemical evaluation

The physicochemical characteristics of the designed chimeras were analyzed using the ProtParam tool on the ExPASy server (https://web.expasy.org/protparam/) [29]. Parameters calculated included molecular weight, theoretical isoelectric point (pI), amino acid composition, extinction coefficient, instability index, aliphatic index, and grand average of hydropathicity (GRAVY). These indices are essential for evaluating protein solubility, stability, and the feasibility of heterologous expression.

To ensure accuracy, analyses were conducted using both the full-length sequences (including the signal peptide) and the mature forms without signal peptides, reflecting the approach followed in the laboratory records. This distinction is critical, since the presence of signal peptides can alter molecular weight, charge distribution, and solubility predictions. Together, these data provided insights into the biochemical properties of the constructs and supported their potential suitability for recombinant expression and purification.

## Results

### Pangenome and reverse vaccinology overview

**Fig. 1:**
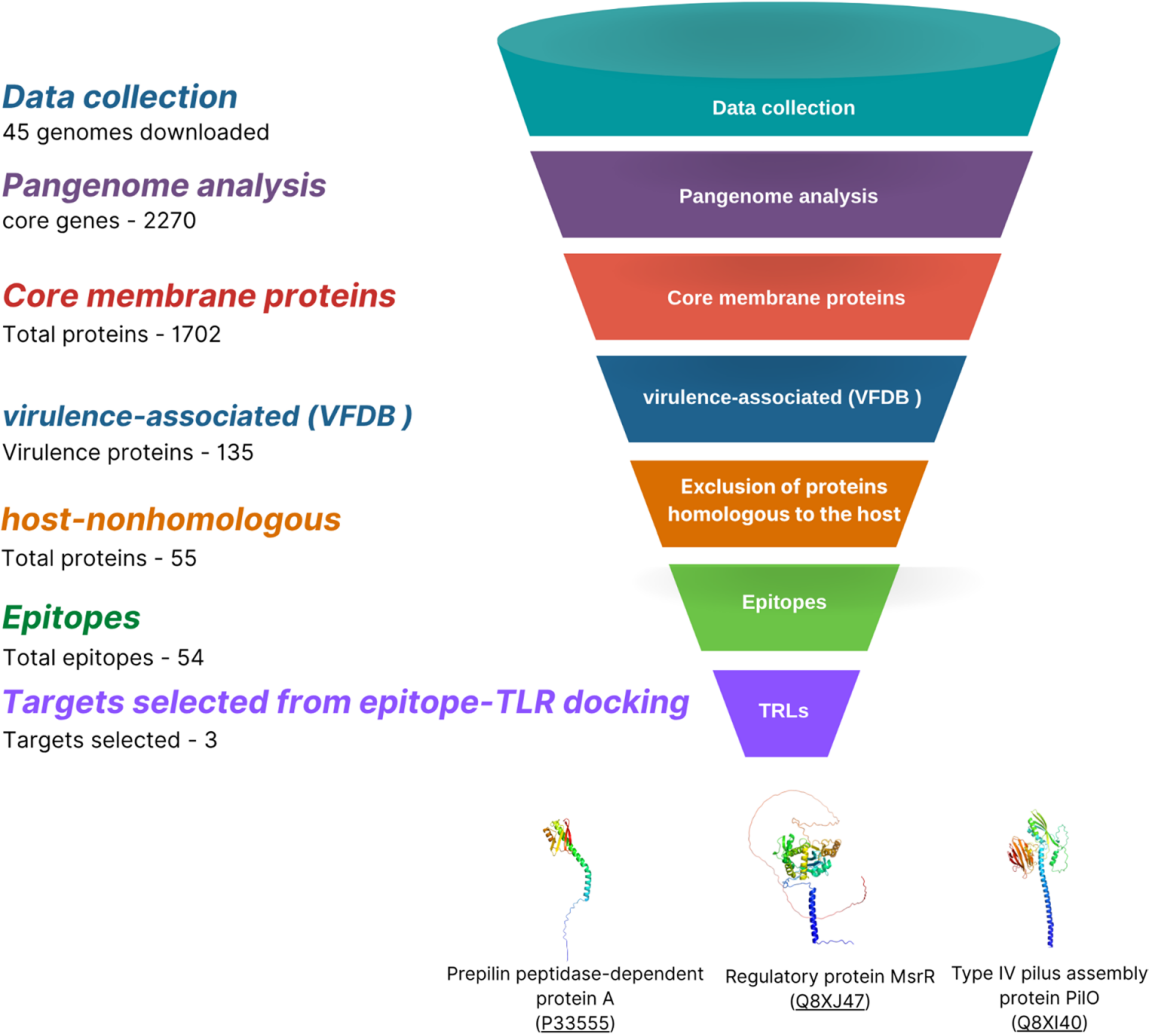
Funnel diagram illustrating the stepwise filtering strategy applied to identify potential vaccine candidates from the *Clostridium perfringens* pangenome. Starting from 8,778 proteins, sequential filters were applied (subcellular localization, transmembrane domain prediction, antigenicity scoring, and virulence factor comparison), resulting in the final selection of three proteins (PilO, Prepilin peptidase-dependent protein A, and MsrR) for multi-epitope chimera construction

Figure 1 outlines the reverse vaccinology pipeline, showing the number of candidates at each filtering step. The process began with the pangenome and culminated in the selection of three final proteins from the core genome, chosen for their high antigenicity and role in virulence. From these proteins, B-cell and MHC-II epitopes were predicted. To identify the most immunogenic candidates, these epitopes were docked against key host immune receptors, including TLR2 (types I and II), TLR4, TLR5, and TLR7. The highest-affinity epitopes were then assembled to create the final multi-epitope chimera.

### Data collection and pangenome analysis

A total of 53 annotated genomes were retrieved from NCBI, of which only 45 were successfully downloaded due to availability issues or the presence of multiple accession numbers for some entries. The pangenome was constructed using these 45 genomes after removal of duplicates, and comprised 8,778 genes, with 2,270 belonging to the core genome and 6,508 to the accessory genome, as shown in Table 1. The core genome includes genes essential for species survival, whereas the accessory genome contains genes that confer strain-specific features, such as environmental adaptation and antimicrobial resistance. Pangenome analysis enables the precise identification of antigenic targets, facilitating the development of multivalent vaccines able to protect against diverse strains of the same pathogen.

**Table 1:** Distribution of the pangenome and core genome size considering different permutations of *Clostridium* genomes

Pangenome fraction	Number of genes
Pangenome (total)	8778
Core genes	2270
Soft core genes	0
Shell genes	1777
Cloud genes	4731

### Immunoinformatics

Translation of the coding sequences yielded a proteome of 8,778 proteins, which were subjected to subcellular localization prediction (PSORTb), transmembrane domain analysis (DeepTMHMM), and genome-wide conservation filtering (≥ 95%). This initial screening produced 1,702 candidate proteins.

Comparison with the VFDB database using the criteria of identity ≥ 30% and bitscore > 100 identified 135 virulence-associated proteins. Subsequent exclusion of homologous proteins to the *G. gallus* proteome (bitscore > 100, identity > 30%) refined the dataset to 55 non-homologous proteins. Among these, classical virulence determinants such as NetB, PFO, NanI-GH33, CPA, CnaA, Tpel, and Zmp were identified with 100% alignment identity, confirming the robustness of the workflow in detecting antigens of known relevance.

Antigenicity screening using VaxiJen (cutoff 0.4) retained a subset of proteins with strong immunogenic potential. This set included both well-established virulence factors (NetB, PFO, CPA) and underexplored targets such as adhesins and cell wall-associated proteins, which were subsequently prioritized for literature and patent database search. Independent evidence from these analyses further supported their involvement in *C. perfringens* pathogenesis and their potential as vaccine or diagnostic candidates.

### Final protein selection and chimera construction

From the non-redundant set of antigenic proteins identified through the *without SignalP* workflow, the three with the highest antigenicity scores, Type IV pilus assembly protein PilO, Prepilin peptidase-dependent protein A, and regulatory protein MsrR, were selected as targets for detailed epitope mapping and multi-epitope chimera construction. The selection of these proteins was guided by a multi-step ranking pipeline combining: (i) antigenicity scoring using VaxiJen (cutoff ≥ 0.4) validated by AntigenPro; (ii) broad conservation across ≥ 95% of the pangenome strains; (iii) surface or transmembrane localization predicted by PSORTb and DeepTMHMM; (iv) confirmed association with virulence functions by VFDB similarity screening (identity ≥ 30%, bitscore > 100); and (v) absence of significant homology to the *G. gallus* proteome (E < 1, bitscore > 100, identity > 30%). Linear B-cell epitopes predicted from these three proteins using IEDB tools were subsequently evaluated through molecular docking (ZDOCK) against *G. gallus* Toll-like receptors (TLR2 type 1 and 2, TLR4, TLR5, and TLR7). The top-ranked epitope clusters exhibited strong receptor-binding affinity, supporting their suitability for downstream multi-epitope chimera design (Table 2).

**Table 2:** *Clostridium perfringens* core-genome surface proteins selected for the chimera construction

Protein	Function	Reference
Type IV pilus assembly protein PilO	Type IV pilus is essential for pathogen adhesion to host cells and is primarily composed of the PilE protein.	[30]
Prepilin peptidase-dependent protein A	An enzyme responsible for cleaving prepilins during pilus assembly and protein secretion.	[31]
Regulatory protein MsrR	MsrR contributes to the development of high-level resistance to β-lactam antibiotics.	[31]

### Chimera construction

The construction of multi-epitope chimeras followed a rigorous approach, starting with the identification of 54 non-redundant epitopes derived from the three most antigenic proteins prioritized in this study (Data Supplementary 2). These epitopes were selected based on antigenicity scores predicted by VaxiJen and validated by AntigenPro, ensuring high immunogenic potential. Molecular docking against TLR4, TLR5, and TLR7 from *G. gallus* enabled the identification of the most promising candidates for incorporation.

**Fig. 2:**
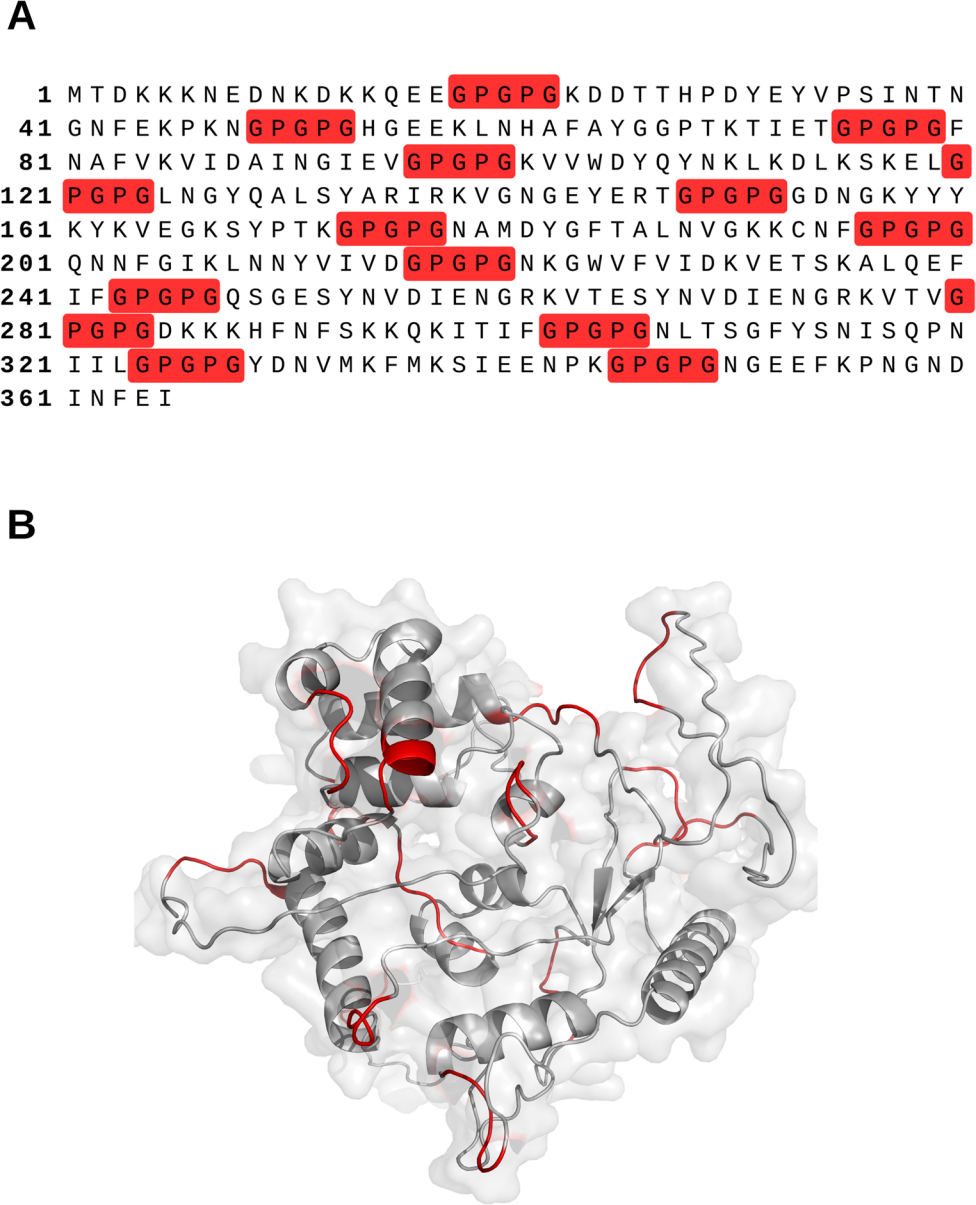
(**A**) primary structure of the designed chimera, with epitopes indicated in white and linkers indicated in red. (**B**) Predicted tertiary structure of the designed chimera modeled using AlphaFold, with linkers indicated in red ribbons, and white regions indicated as white

A total of 60 chimeras were generated, exploring alternative structural configurations and including histidine tags to facilitate downstream purification, and the best one (Fig. 2) was chosen based on the VaxiJen webserver. To guarantee efficient antigen exposure, hydrophilicity analyses using IEDB were performed, confirming that immunodominant regions were oriented on the protein surface. Chimera assembly employed GPGPG linkers, preserving epitope integrity and minimizing steric hindrance or undesired interactions.

Final molecular docking analyses demonstrated high binding affinity of the chimeras to the TLRs, highlighting their significant potential to elicit strong immune responses and supporting their use as promising vaccine constructs against *C. perfringens*.

## Discussion

The methodological strategy adopted in this study represents an advancement compared to the approaches traditionally applied to the development of vaccines against *C. perfringens* associated with avian necrotic enteritis. While most previous studies have focused on antigens such as alpha-toxin (CPA) and NetB toxin, the main virulence factors recognized in the pathogenesis of the disease [3, 4]. The present study proposed a pangenome-based approach guided by reverse vaccinology, focusing on non-toxic antigens that are highly conserved among pathogenic strains.

The pipeline developed in this study was designed to maximize the specificity, genomic conservation, and immunological potential of vaccine candidates. Starting from 8,778 proteins derived from the pangenome of 45 *C. perfringens* genomes, successive filters were applied, including subcellular localization (PSORTb), transmembrane domain prediction (DeepTMHMM), and ≥ 95% conservation, yielding 1,702 proteins potentially exposed to the host immune system. Comparison with the Virulence Factor Database (VFDB) under criteria of identity ≥ 30% and bitscore > 100 identified 135 virulence-associated proteins. Subsequent exclusion of homologs to the *G. gallus* proteome (E < 1, bitscore > 100, identity > 30%) further refined the dataset to pathogen-specific candidates, which were then screened for antigenicity using VaxiJen (cutoff ≥ 0.4). This process yielded a subset of highly antigenic and non-allergenic proteins, from which the top three PilO, Prepilin peptidase-dependent protein A, and MsrR were prioritized for detailed epitope mapping and multi-epitope chimera design.

The antigenicity of the selected proteins was evaluated using the VaxiJen v2.0 program [20], which allows the estimation of immunogenic potential based on physicochemical properties independent of sequence alignment. The results indicated that the three prioritized proteins exhibited higher predicted values than those observed for classical *C. perfringens* antigens. PilO showed a predicted value of 0.85, Prepilin peptidase A had the highest score (0.99), and MsrR reached 0.80, all above the 0.5 threshold considered indicative of antigenicity. In contrast, proteins traditionally associated with necrotic enteritis, such as NetB (0.62) and CPA (0.57), displayed lower values. These findings suggest that, although they are not toxins, the structural and regulatory proteins identified in this study possess antigenic potential comparable to or greater than classical virulence factors. This reinforces the rationale for prioritizing non-toxic, conserved, and functionally relevant targets capable of eliciting a protective immune response without the risks associated with cytotoxic antigens. This is consistent with previous findings showing that type IV pilus-associated proteins contribute to adhesion, colonization, and biofilm formation, highlighting their relevance as vaccine targets. Additionally, non-toxin proteins involved in adhesion and tissue interaction have been shown to significantly contribute to necrotic enteritis pathogenesis [32, 33].

The pangenomic analysis was conducted using 45 complete *C. perfringens* genomes, carefully selected as, at the time of download, they were the only genomes available in the NCBI database with avian necrotic enteritis (NE) clearly characterized in their metadata descriptions. This filtering ensured that the analyzed dataset represented exclusively isolates associated with the disease, avoiding the inclusion of strains of environmental, food, or unspecific clinical origin that could introduce functional noise in the prediction of antigens related to NE pathogenesis. Thus, the resulting pangenome reflects the gene repertoire characteristic of pathogenic lineages, thereby increasing the biological relevance of the identified vaccine targets. This is particularly relevant considering the high genomic plasticity of *C. perfringens*, in which a large proportion of accessory genes, including toxin genes, are variably distributed among strains, reinforcing the importance of targeting conserved proteins for broader protection [32].

This methodological combination contrasts with previous studies that applied reverse vaccinology in a more limited manner. For example, Qin et al. [34] analyzed 15 bovine genomes of *C. perfringens* type A and applied structural modeling using AlphaFold2, along with conformational epitope prediction through DiscoTope-3.0 [35], identifying potentially antigenic surface proteins. However, the focus remained on structural characterization and B-cell epitope prediction, without integrating immune receptor interaction analyses, although their approach did include a pangenomic evaluation. In contrast, the present study, by incorporating docking with multiple TLRs (2, 4, 5, and 7) as well as genomic conservation (≥ 95%) and host homology filters, substantially expands the immunological and comparative dimensions of antigen selection.

Recent studies reinforce the applicability of multi-epitope vaccines (MEVs) against *C. difficile* [36] reported a 66–amino acid construct based on the ABC-type transport system protein, validated in silico through structural modeling, docking with TLR3, and 100-ns molecular dynamics simulations that demonstrated high structural stability (RMSD ≈ 0.1 Å) and strong binding affinity. Immune simulations predicted robust B- and T-cell responses, with elevated levels of IFN-γ and IL-2, supporting the potential of the construct as a safe and effective prophylactic. Previous studies [7, 37, 38] complement this perspective by highlighting non-toxic targets, such as flagellar and spore proteins (e.g., FliD and CdeC), suggesting that rational combinations of epitopes could broaden protective coverage. Taken together, these findings indicate that MEVs for *C. difficile* are rationally designed, structurally stable, and biologically promising, guiding future strategies for formulation and preclinical evaluation.

The screening performed using VaxiJen 2.0 identified 24 proteins with antigenicity values above 0.6, indicating a highly promising set for vaccine exploration. The highest scores were observed for Prepilin peptidase–dependent protein A (0.9940), Type IV pilus assembly protein PilO (0.8546), and Regulatory protein MsrR (0.8035), followed by Type II secretion system protein (0.7943) and Pilin isopeptide linkage domain–containing protein (0.7261). The average antigenicity score of the set was 0.69, reflecting an overall immunogenic profile.

Although the chimera design prioritized the three most relevant antigens (PilO, Prepilin, and MsrR), the remaining proteins also exhibit potential for individual or complementary application. For instance, adhesion-related proteins such as the Cna protein B-type domain–containing protein (0.7145) and the probable collagen adhesin (0.7183) [39, 40], could be used as isolated recombinant subunits to elicit humoral responses against colonization mechanisms. Other proteins associated with secretion and cell wall modification, such as serine proteinase (0.6970) and exo-α-sialidase (0.6287) (serine1, serine2, serine3), could be incorporated into multicomponent vaccines or presented in alternative structural formats. Thus, the selection of these 24 proteins provides a diverse panel of surface and secreted antigens with potential for both individual and integrated applications, reinforcing the versatility of the proposed methodology.

For chimera construction, the GPGPG linker was selected because it favors the proper presentation of epitopes to helper T cells, thereby promoting a robust and balanced immune response. Recent studies highlight that this linker provides structural flexibility and stability to the chimera, maintaining adequate epitope exposure and preventing conformational interference. This strategy has been widely applied in multi-epitope vaccines against various pathogens, demonstrating efficacy in enhancing antigen immunogenicity [41–44].

Similarly, Heidarpanah et al. [44] examined *C. perfringens* strains for five surface proteins exclusive to pathogenic chicken isolates, including prepilin-like proteins associated with type IV pilus assembly, confirming their immunogenicity but without integrating pangenomic screening or epitope prediction. Moreover, recent genomic analyses have shown that type IV pilus assembly proteins, such as PilO, PilM, and PilT, are widely conserved among *C. perfringens* isolates associated with necrotic enteritis [44, 45], reinforcing that conserved structural antigens are promising components in multivalent vaccine formulations.

On the other hand, the present study incorporated molecular docking analyses using the ZDOCK server,, allowing the evaluation of interactions between selected epitopes and multiple Toll-like receptors (TLR2, TLR4, TLR5, and TLR7) of *G. gallus*. This structural stage was extended in previous studies [46], which considered only TLR7, thereby significantly expanding the prediction of both innate and adaptive immune activation. In addition, three-dimensional modeling with AlphaFold [28] and physicochemical analysis with ProtParam [47] ensured the stability and structural feasibility of the multi-epitope construct.

While experimental quadrivalent vaccines already achieved approximately 77–80% reductions in intestinal lesions by combining toxins and adhesins (NetB, CPA, CnaA, and metalloproteases) [48], the formulation proposed in this study stands out by targeting non-toxic, conserved, and functionally synergistic proteins. PilO and prepilin act together in the assembly of type IV pili, which are essential for bacterial adhesion and motility [49, 50], whereas MsrR is involved in cell wall regulation and β-lactam resistance [31]. This association between offensive (adhesion) and defensive (cell integrity) mechanisms broadens the potential spectrum of protection and reduces the risk of adverse effects, as also observed in non-toxigenic models of *C. difficile* [7, 8]. By focusing on these non-toxin proteins, the vaccine strategy proposed here aims to address both the bacterial persistence mechanisms and enhance immunity without relying solely on toxin neutralization, offering a potentially safer and more comprehensive solution [51]. In this context, it is important to note that excessive inflammatory responses induced by virulent strains may contribute to tissue damage and disease progression, suggesting that targeting non-toxin antigens involved in colonization and persistence may provide a more balanced and effective immune response [52].

To better contextualize the biological relevance of the selected targets, it is important to consider their roles in both colonization and bacterial persistence. Proteins associated with type IV pili, such as **PilO** and **prepilin peptidase-dependent protein A**, are directly involved in adhesion to host tissues and are therefore highly relevant for preventing the initial stages of *C. perfringens* colonization [39]. In contrast, **MsrR** is not directly implicated in adhesion but plays a role in cell wall homeostasis and resistance to environmental stressors. Although immune responses targeting MsrR may not prevent bacterial attachment, they could impair bacterial fitness and survival within the host, contributing indirectly to infection control. This combined targeting strategy, involving both colonization-related and persistence-associated proteins, may enhance the overall effectiveness of the proposed vaccine. Nevertheless, additional virulence factors directly involved in host interaction, such as **collagen adhesion proteins (CNA)** and **fibrinogen-binding proteins**, represent promising candidates for future investigation and may further improve vaccine performance.

Another key difference lies in the genomic and immunological scope. The use of 45 genomes allowed the identification of targets present in most strains associated with necrotic enteritis, overcoming the limitation of genetic variability reported in previous pangenome analyses [53]. Moreover, the prediction of B-cell and MHC-II epitopes, rather than only linear epitopes, provides greater potential for the simultaneous induction of humoral and cellular immune responses a crucial element for the establishment of long-term immunological memory [44, 54].

Thus, when compared with experimental toxin-based formulations [34, 48] and genomic analyses lacking structural validation [55, 56], the methodology proposed in this study suggests greater safety, antigenic breadth, and immunological predictability, although it still requires in vivo validation. The integration of pangenomics, immunoinformatics, and structural modeling aligns with recent initiatives in *Klebsiella pneumoniae* [57] and *Haemophilus influenzae* [33], establishing itself as a next-generation strategy for the rational development of recombinant vaccines against multivirulent pathogens.

This study presents some inherent limitations associated with in silico vaccine design. First, conformational B-cell epitopes were not evaluated, as this analysis is not meaningful in multi-epitope vaccines (MEVs) where protein fragmentation disrupts tertiary structure, and only linear epitopes remain accessible for immune recognition. Second, *G. gallus* lacks a fully characterized MHC-II allele repertoire, and available predictors are primarily trained on mammalian datasets, limiting the precision of host-specific epitope affinity estimations. Third, molecular docking to Toll-like receptors (TLRs) provides valuable insights into receptor–ligand interactions but does not substitute for adjuvant design or formulation optimization, which must be experimentally defined. These caveats highlight the need for empirical validation to confirm the immunogenicity and protective efficacy predicted computationally.

Although the in silico predictions establish a robust theoretical foundation for the multi-epitope chimera, rigorous empirical validation is essential to confirm its prophylactic efficacy. We propose an experimental framework involving recombinant expression and purification of the construct, followed by formulation with appropriate adjuvants to enhance immunogenicity, as commonly employed in subunit and multi-epitope vaccine platforms [58]. Subsequent in vivo evaluation using a well-established chicken model of necrotic enteritis will enable assessment of the induced adaptive immune response, including quantification of antigen-specific serum IgY and mucosal IgA levels, which are key correlates of protection in avian systems [59].

Primary efficacy endpoints may include reduction of intestinal lesion scores, a gold-standard metric for necrotic enteritis severity, alongside quantification of bacterial load in the gastrointestinal tract and evaluation of zootechnical performance parameters such as weight gain and survival [60]. Similar experimental designs in multi-epitope and recombinant vaccine studies have demonstrated that effective immunization leads to enhanced humoral responses, reduced pathogen burden, and improved clinical outcomes in poultry infection models [61]. To our knowledge, this experimental design provides a robust framework for the future evaluation of the proposed chimera as a protective vaccine candidate.

In summary, the results reinforce that the combination of epitopes from conserved structural proteins PilO, prepilin peptidase A, and MsrR may provide broader and safer protection than vaccines based on isolated toxins, with the potential to reduce both intestinal colonization and pathogen persistence. Although practical efficacy still depends on experimental validation, the in silico evidence indicates a highly stable, immunogenic, and low-risk construct capable of contributing to the sustainable control of avian necrotic enteritis in the post-antibiotic era.

## Conclusion

In this study, we applied a pangenome-guided reverse vaccinology approach integrated with immunoinformatics to identify conserved and antigenic proteins of *C. perfringens* associated with avian necrotic enteritis. Through sequential filtering of 45 genomes, we reduced an initial dataset of 8,778 proteins to three prioritized candidates: the type IV pilus assembly protein PilO, the prepilin peptidase-dependent protein A, and the regulatory protein MsrR. These proteins exhibited complementary roles in adhesion, pilus biogenesis, and cell wall homeostasis, reinforcing their relevance as potential vaccine targets. Epitope prediction, structural modeling, and molecular docking against chicken Toll-like receptors confirmed the strong immunogenic potential of the designed multi-epitope chimera, with favorable physicochemical properties that support its feasibility for recombinant expression.

Building on these findings, the identification of PilO, Prepilin peptidase A, and MsrR underscores the importance of non-toxin antigens as promising vaccine candidates against *C. perfringens*. Because they are conserved across pathogenic strains and functionally complementary, they represent attractive targets for broad-spectrum protection. By extending beyond toxin-based formulations, this strategy highlights the potential of non-toxic vaccine approaches to enhance the breadth and durability of protection against necrotic enteritis in poultry, complementing or even surpassing traditional toxin-focused designs. Future work should focus on experimental validation of the selected epitopes and chimeras in vitro and in vivo to confirm their protective efficacy and assess their potential for commercial application.

## Supplementary Information

Below is the link to the electronic supplementary material.

Supplementary Material 1

Supplementary Material 2

Supplementary Material 3
